# Correlation between patients’ anatomical characteristics and interfractional internal prostate motion during intensity modulated radiation therapy for prostate cancer

**DOI:** 10.1186/s40064-015-1382-z

**Published:** 2015-10-06

**Authors:** Shintaroh Maruoka, Yasuo Yoshioka, Fumiaki Isohashi, Osamu Suzuki, Yuji Seo, Yuki Otani, Yuichi Akino, Yutaka Takahashi, Iori Sumida, Kazuhiko Ogawa

**Affiliations:** Department of Radiation Oncology, Osaka University Graduate School of Medicine, 2-2 Yamadaoka, Suita, Osaka, 565-0871 Japan; Kaizuka City Hospital, 3-10-20 Hori, Kaizuka, Osaka, 597-0015 Japan

**Keywords:** Prostate cancer, Internal motion, Interfractional motion, Megavoltage cone beam computed tomography, Body mass index

## Abstract

Intensity modulated radiation therapy (IMRT) is one of a standard treatment for localized prostate cancer. Although lower complication is expected for smaller target margin, determination of optimal margin is important. For bony-structure based registration, internal prostate motion is the main factor determining the margin from clinical target volume to planning target volume. The purpose of this study was to measure interfractional internal motion of the prostate and to identity the factors which enlarge or reduce the margin, with special focus on patients’ anatomical characteristics. The 586 image sets of 16 patients acquired with megavoltage cone beam computed tomography were analyzed. For each patient, prostate shift in three directions was recorded for each fraction to calculate the required margin. Correlations between these values and patients’ anatomical characteristics were evaluated. The posteriorly required margin correlated positively with rectal volume and rectal mean area (p = 0.015 and p = 0.008), while random error in lateral, craniocaudal and anteroposterior direction correlated negatively (p = 0.014, 0.04 and 0.0026, respectively) with body mass index (BMI). In addition to the previously identified factor of distended rectum, BMI was newly identified as another significant factor influencing interfractional internal prostate motion.

## Background

External beam radiotherapy (EBRT) is a main therapeutic modality for localized prostate cancer (Mohler et al. 2012). Some randomized studies have demonstrated the efficacy of dose-escalated EBRT for the treatment of localized prostate cancer (Dearnaley et al. 2007; Al-Mamgani et al. 2008; Kuban et al. 2011), but such efficacy often involves a trade-off in the form of heightened rectal toxicity. Intensity modulated radiation therapy (IMRT) is an improved version of EBRT that produces a steep dose gradient between the prostate and the surrounding risk organs such as rectum and bladder. EBRT using IMRT can deliver a higher dose to the prostate while keeping the dose to risk organs low, but the steep dose gradient results in a higher risk of setup error and internal prostate motion than with previous procedures. In fact, the National Comprehensive Cancer Network (NCCN) guidelines require image guided radiation therapy (IGRT) if the prescription dose is 78 Gy or more.

The common procedures for image guidance include trans-abdominal ultrasonography (US), in-room helical computed tomography (CT), on-board cone beam CT (megavoltage or kilovoltage), and electric portal imaging devices (EPID) with or without implanted fiducial markers (Stephans et al. 2010; Soete et al. 2008). Although these modalities are helpful for accurate patient set up, each procedure has some disadvantages. The accuracy of trans-abdominal US is affected by the technical capability of the performer, and organ displacement may occur due to image acquisition (Soete et al. 2008). Image guidance by in-room or cone beam CT requires extra medical staff and generates increased radiation exposure for the patients, especially for prostate based registration. EPID is an older technique but it is still widely used for bony structures-based registration. Although target position can be identified with fiducial markers, implantation of fiducial markers is invasive. Although the prostate-based registration may preferable for IMRT, bony structures-based registration is still majority of the image-guided radiotherapy because of its simplicity, convenience, and less invasiveness. For bone-matching registration, internal prostate motion is the main factor which defines the margin from clinical target volume (CTV) to planning target volume (PTV). The purpose of this study was to measure interfractional internal motion of the prostate and to identify the factors which enlarge or reduce such a margin, with special focus on patients’ anatomical characteristics.

## Methods

### Patients

This study was performed with permission of the Institutional Review Board of our hospital. Between October 2010 and May 2011, 16 patients with localized prostate cancer participated in this clinical trial. All of them were informed of this clinical trial and agreed by document to the participation in this study. All of them completed the study regimen. The patient distribution for T-stage was T1: T2: T3 = 5: 7: 4, for the Gleason score ≤6: 7: 8≤ = 6: 4: 6, for the pretreatment prostate-specific antigen level <10 ng/ml: 10–20 ng/ml: 20 ng/ml< = 7: 5: 4, and for low: intermediate: high risk = 3: 6: 7. The definitions of these factors were derived from the NCCN guidelines. Table 1 shows the patients’ anatomical characteristics which we considered to be candidates for affecting interfractional internal motion of the prostate.

**Table 1:** Patients’ anatomical characteristics

Variables	Means (range)
Age (years old)	71.5 (60–81)
BMI (kg/m^2^)	23.8 (14.8–30.7)
Prostate volume (cc)	22.5 (14.2–51.1)
Bladder volume (cc)	144.1 (34.7–645.1)
Rectal volume (cc)^a^	42.0 (28.9–55.2)
Rectal mean area (cm^2^)^b^	5.86 (4.12–7.46)

^a^Rectal volume was measured from the 2-cm above the prostate base level to the 2-cm below the prostate apex level

^b^Rectal mean area was calculated as rectal volume divided by its length in craniocaudal direction

### Radiotherapy, acquisition of registry image, and measurement of prostate shift

Oncor Impression PLUS™ with a megavoltage cone beam CT (MV-CBCT) system (MVisionTM; Siemens Medical Solutions, Concord, CA, USA) was used. Patients emptied their rectum in the morning of CT simulation as well as their bladder 30 min before CT simulation. Patients were immobilized in supine position using Vac-Lok™ Cushions (CIVCO Medical Solutions, Orange City, IA, USA). Contouring was performed by an experienced radiation oncologist. CTV was defined as the prostate plus the medial part of seminal vesicles, and PTV as CTV plus 5 mm margin in all directions. The prescription dose was 70 Gy/35 fractions for low risk group and 74 Gy/37 fractions for intermediate or high risk group, respectively. The dose was normalized to cover 95 % of PTV with prescribed dose. Dose-volume constraints for risk organs were: rectum V45Gy <35 %, V65Gy <17 %, bladder V40Gy <50 %, V65Gy <25 %, femoral head Dmax <50 Gy, and small intestine Dmax <60 Gy. All the patients were treated with step-and-shoot IMRT.

We used a unique technique, described elsewhere (Akino et al. 2012), for image guidance that involved MV-CBCT and its characteristic treatment planning method. The dose for image guidance had been assigned as 15 monitor units (MU), which was considered part of the treatment dose. The normal step-and-shoot IMRT beam arrangements were then made by using the reoptimization process. As a typical example, 2 Gy per fraction comprised the daily MV-CBCT dose of 0.1 Gy (corresponding to 15 MU) and the usual IMRT beam dose of 1.9 Gy. Increased MU MV-CBCT can be used to identify soft-tissue structure such as the prostate (Morin et al. 2006), although its image quality is second to that of CT using kV energy (Morrow et al. 2012). Before the daily administration of step-and-shoot IMRT, 15 MU-MV-CBCT was acquired and the patient’s position was three-dimensionally corrected based on bone structure by radiotherapists with or without aid of the auto-correcting function. If rectal gas was apparent on MV-CBCT, a soft catheter was used for gas suction. No correction based on prostate matching was performed before the actual IMRT beam delivery. All the MV-CBCT images were then reviewed retrospectively by radiation oncologists in order to evaluate shift of the prostate from the original position determined at the CT simulation stage and measured in three directions (lateral, craniocaudal and anteroposterior). The extent of shift was measured when the radiation oncologists had determined the optimal position. This was done by overlaying the prostate on MV-CBCT images onto the contour of the prostate delineated at the treatment planning using CT simulation images. Deformation or rotational shift of the prostate was not taken into consideration.

### Data analysis and statistics

Correlations between characteristics shown in Table 1 and the mean prostate shift, the standard deviation (SD) of prostate shift, and the “required margin”, were statistically examined. In this study, we defined “required margin” as a margin from CTV to PTV, satisfying the condition: “interfractional motion of CTV in each direction doesn’t exceed PTV with a probability of 95 %” in each patient. Assuming a normal distribution of prostate shift, the “required margin” was calculated as follows: “Required margin” = (the mean of prostate shift) + 1.96 × (SD of prostate shift), in every direction for each patient. The respective correlations between patients’ anatomical characteristics and the required margin in six directions (left, right, cranial, caudal, anterior and posterior) or the mean and SD of prostate shift in three directions (lateral, craniocaudal and anteroposterior) were evaluated by means of linear regression analysis. A p value of <0.05 was considered statistically significant. JMP software version 9.0.2 (SAS Institute Inc. Cary, NC, USA) was used in this study.

## Results

**Fig. 1:**
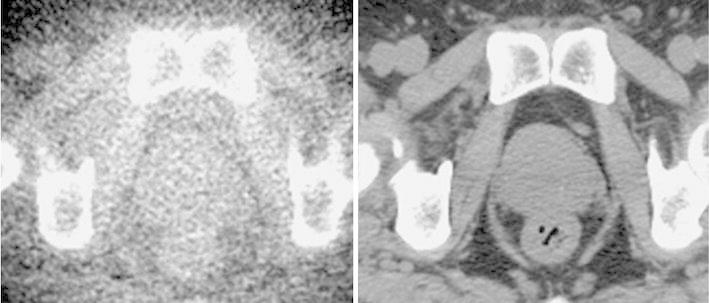
A sample of MV-CBCT image (*left*) and the corresponding kilovoltage CT image at the treatment planning (*right*)

**Fig. 2:**
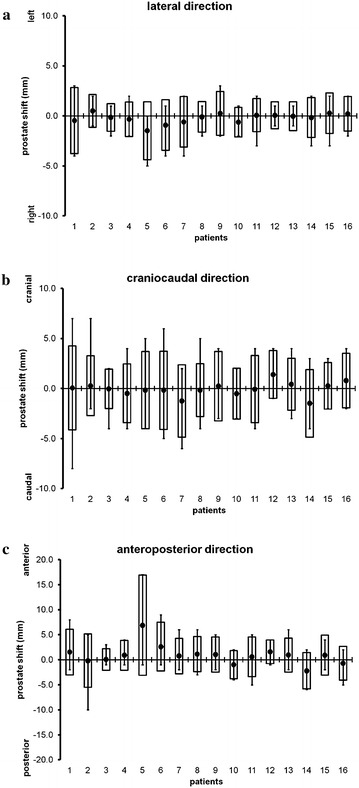
Three dimensional shifts of the prostate in individual patients. *Lines* indicate the range of prostate shift during 35–37 fractions for each patient. *Dots* show the mean of prostate shift from the original location determined by CT simulation. *Columns* represent the mean ± 1.96 SD, which indicate the range where the prostate is located with a probability of 95 % with an assumption of a normal distribution. **a**–**c** Lateral, craniocaudal and anteroposterior shift of prostate, respectively

A total of 586 MV-CBCT image sets were acquired from the 16 patients. Prostate position was clearly identified in 74–97 % (median 86 %) of the image sets for each patient (513 image sets in total, 88 % of all image sets). A sample of MV-CBCT image and the corresponding kilovoltage CT image at the treatment planning is shown in Fig. 1. Rests of the images were not of enough quality to identify position of prostate. Position of bone structure was clearly identified in 92–100 % per patient (altogether 573 sets, 98 % of the total). Three-dimensional shift (lateral, craniocaudal and anteroposterior) of the prostate is shown in Fig. 2a–c, respectively, where the means, ranges and “required margins” are shown. For Fig. 2a–c, positive values of Y-axis represent the shift in left, cranial, and anterior directions, respectively. Note that the positive and negative values represent the left/cranial/anterior and the right/caudal/posterior motion, respectively. The mean and range of “required margin” in each direction is shown in Table 2. Correlations between “required margin” in each direction and patients’ anatomical characteristics are shown in Table 3. The margin in the posterior direction correlated positively with rectal volume and rectal mean area (p = 0.015 and p = 0.008, respectively). The margin in the anterior and right directions correlated negatively with BMI (p = 0.0047 and 0.0016, respectively), which indicated that a larger BMI correlated with a smaller margin. No statistically significant correlations were found for the other directions. Next, the mean (systemic error) and the SD (random error) of daily prostate shift were investigated statistically in terms of their correlations with patients’ anatomical characteristics (Tables 4, 5). The systematic error in the lateral directions correlated positively and anteroposterior directions correlated negatively with BMI (p = 0.0008 and p = 0.028, respectively). The random error in all directions also correlated negatively with BMI (lateral, craniocaudal and anteroposterior: p = 0.014, 0.04 and 0.0026, respectively).

**Table 2:** The mean and range of required margin

Dimension	Required margin
	Mean (range)
Lateral (mm)	
Left	1.7 (0.9–2.8)
Right	1.9 (1.2–4.4)
Craniocaudal (mm)	
Cranial	3.2 (1.9–4.3)
Caudal	3.1 (1.0–4.9)
Anterioposterior (mm)	
Anterior	4.6 (1.4–17.0)
Posterior	3.1 (0.8–6.9)

**Table 3:** Correlation between required margin and patients’ anatomical characteristics

	Lateral		Craniocaudal		Anteroposterior	
	Left	Right	Cranial	Caudal	Anterior	Posterior
Age (years old)	p = 0.39	p = 0.95	p = 0.40	p = 0.84	p = 0.80	p = 0.46
BMI (kg/m^2^)	p = 0.74	p = 0.0016^††^	p = 0.36	p = 0.09	p = 0.0047^††^	p = 0.72
Prostate volume (cc)	p = 0.87	p = 0.91	p = 0.93	p = 0.19	p = 0.71	p = 0.46
Bladder volume (cc)	p = 0.15	p = 0.95	p = 0.36	p = 0.35	p = 0.29	p = 0.41
Rectal volume (cc)	p = 0.18	p = 0.61	p = 0.72	p = 0.70	p = 0.96	p = 0.015^†^
Rectal mean area (cm^2^)	p = 0.23	p = 0.92	p = 0.57	p = 0.97	p = 0.70	p = 0.008^†^

^†^Significantly positive correlation, ^††^significantly negative correlation

**Table 4:** Correlation between systematic error (mean of prostate shift) and patients’ anatomical characteristics

	Lateral	Craniocaudal	Anteroposterior
Age (years old)	p = 0.75	p = 0.78	p = 0.93
BMI (kg/m^2^)	p = 0.0008^†^	p = 0.42	p = 0.028^††^
Prostate volume (cc)	p = 0.96	p = 0.29	p = 0.68
Bladder volume (cc)	p = 0.45	p = 0.21	p = 0.17
Rectal volume (cc)	p = 0.28	p = 0.90	p = 0.68
Rectal mean area (cm^2^)	p = 0.55	p = 0.73	p = 0.93

^†^Significant positive correlation, i.e. systematic error tends to be left/cranial/anterior as parameters rise, ^††^significant negative correlation, i.e. systematic error tends to be right/caudal/posterior as parameters rise

**Table 5:** Correlation between random error (SD of prostate shift) and patients’ anatomical characteristics

	Lateral	Craniocaudal	Anteroposterior
Age (years old)	p = 0.71	p = 0.44	p = 0.99
BMI (kg/m^2^)	p = 0.014^††^	p = 0.04^††^	p = 0.0026^††^
Prostate volume (cc)	p = 0.94	p = 0.21	p = 0.90
Bladder volume (cc)	p = 0.67	p = 0.79	p = 0.44
Rectal volume (cc)	p = 0.95	p = 0.55	p = 0.41
Rectal mean area (cm^2^)	p = 0.78	p = 0.72	p = 0.22

^††^Significantly negative correlation

## Discussion

A significant number of studies have cautioned about the problem of interfractional internal motion of the prostate, especially in the anteroposterior direction, during prostate cancer treatment using EBRT (Bylund et al. 2008; Snir et al. 2011). Because the volume of the rectum easily changes depending on its contents of gas and feces, managements of the anteroposterior motion of the prostate during EBRT is of utmost importance in terms of two aspects: tumor control and late toxicity. De Crevoisier et al. (2005) showed that for intermediate and high risk prostate cancer patients, a larger average cross-sectional rectal area (defined as rectal volume divided by its length) was associated with a higher risk of biochemical failure. This can be explained by the fact that a large rectal volume represents a high potential of posterior displacement of the prostate, which might lead to underdosing the tumor if combined with a small posterior margin. The association may also be based on the fact that many prostate cancer cells are often located at the posterior site within the prostate gland (Chen et al. 2000). Interestingly, De Crevoisier’s study also showed that the assumption of a large rectum for planning CT was associated with reduced rectal adverse events. Reddy et al. used B-mode trans-abdominal US to show that the prostate tended to move posteriorly when the rectal volume on treatment planning CT was large, while it tended to move anteriorly when the rectal volume was small (Reddy et al. 2009). In support of these studies, our results also showed a significant correlation between the required posterior margin and the rectal volume or the rectal mean area. However, we wish to emphasize that the absolute value of the required margin was larger in the anterior direction (mean 4.6 mm) than in the posterior direction (mean 3.1 mm), which may well be attributable to the thorough gas suction or encouraging evacuation before planning CT used in our study. Although a statistical correlation could be found in the posterior direction, the absolute influence (required margin) could be kept low by keeping the absolute rectal volume or mean area low. Another finding of this study deals with BMI. An increase in setup errors in EBRT for overweight patients has been reported in the literature (Millender et al. 2004). In addition, a high frequency of biochemical failure for overweight patients was also reported (Geinitz et al. 2011). In contrast, patients treated with permanent brachytherapy did not show any significant differences in outcome between normal and overweight patients (Merrick et al. 2005), which might be explained by the fact that brachytherapy was not affected by daily setup errors although caution is needed when using EBRT for overweight patients (Wong et al. 2009). Although correlations between daily setup errors and the effect of obesity have been thoroughly studied, only a few studies have investigated correlation between BMI and prostate internal motion. Thompson et al. (2011) showed that intrafractional prostate motion did not increase in overweight patients, but rather decreased in the craniocaudal direction. Since our study showed a negative correlation between BMI and extent of prostate intefractional internal motion, high BMI may thus be a factor that reduces the margin derived from prostate internal motion. Male obesity tends to involve fat tissue at the trunk compared to female obesity (Chambers et al. 2010), so that we often encounter such patients with extra fat at the abdomen. This means that, when they are in the supine position, the fat may compress the prostate into the caudal and dorsal direction. This may be the reason why there were fewer random errors in the patients with high BMI in our study, as well as account for finding of Thompson’s study that the intrafractional craniocaudal motion could be smaller for the overweight patients (Thompson et al. 2011). This study also showed a negative correlation between BMI and the right margin, whereas no correlation was shown between BMI and the left margin. This result is assumed to be associated with the positive correlation between BMI and systematic error to the left direction (Table 4). Although we failed to explain the reason of this correlation, we thought the clinical importance of this discrepancy would be limited, because the motion in the lateral direction was the smallest in the three directions, with the absolute value of lateral systematic error as small as 0.4 mm as the mean, with the range from 0.5 mm to 1.5 mm.

This study has several limitations. First, although the total number of images evaluated in this study was as many as 513, we examined only 16 patients. There remains a possibility that these 16 patients were not representative of normal population, which may weaken our conclusion. Further researches with enough statistical power are warranted to establish the definite relationship between BMI and random error. Second, we investigated only the interfractional motion of the prostate, since an examination of intrafractional motion was beyond the scope of this study, which is similar to the feature of other studies of interfractional prostate motion. The required margin as defined in this study can therefore not be utilized as an adequate margin as such in clinical settings. In addition, deformation or rotational movement was not dealt with in this study. This would indicate that the findings obtained from this study would be of only limited value because they become irrelevant for prostate-matching IGRT. However, as prostate-matching procedure requires sophisticated liniac systems and human resources, we believe bone-matching IGRT is still worth trying taking into consideration its ease of application and that paying attention to BMI is important.

## Conclusions

The use of 15 MU MV-CBCT before every fraction of IMRT allowed us to evaluate prostate shift on a daily basis and required margin. Patients with distended rectum on planning CT needed a more posterior margin, as was also suggested by previous studies. We found another factor influencing interfractional internal prostate motion, that is, BMI. Interestingly, a high BMI was associated with a smaller motion or a smaller margin, which is quite contrary to the belief of the preceding radiotherapy era that high BMI is associated with a larger setup error. When we treat patients with low BMI or with distended rectum on planning CT, setting a relatively larger margin, especially in anteroposterior direction should be considered, as long as bone-matching IGRT is used. In such patients, the benefit of prostate-matching IGRT would be greater, while brachytherapy might be considered as an alternative treatment option. In other words, prostate-matching IGRT using fiducial markers or some other, similar technique for the treatment for patients with high BMI or whose rectum is not distended may yield relatively small gains.

## Abbreviations

EBRT: external beam radiotherapy
IMRT: intensity modulated radiation therapy National Comprehensive Cancer Network (NCCN)
IGRT: image guided radiation therapy
US: ultrasonography
CT: computed tomography
EPID: electric portal imaging devices
CTV: clinical target volume
PTV: planning target volume
MV-CBCT: megavoltage cone beam CT
MU: monitor units
SD: standard deviation
BMI: body mass index
